# ﻿*Isodonxiaoluzhiensis* (Lamiaceae, Nepetoideae), a new species from Yunnan, southwest China

**DOI:** 10.3897/phytokeys.237.117071

**Published:** 2024-01-24

**Authors:** Shi-Gang Li, Qiang-Chun Huang, Shao-Yun Liu, Chun-Lei Xiang, Huan-Chong Wang

**Affiliations:** 1 School of Ecology and Environmental Science, Yunnan University, Kunming 650091, Yunnan, China Yunnan University Kunming China; 2 Southwest United Graduate School, Kunming 650092, Yunnan, China Southwest United Graduate School Kunming China; 3 CAS Key Laboratory for Plant Diversity and Biogeography of East Asia, Kunming Institute of Botany, Chinese Academy of Sciences, Kunming 650201, China Kunming Institute of Botany, Chinese Academy of Sciences Kunming China; 4 Herbarium of Yunnan University, Kunming 650091, Yunnan, China Herbarium of Yunnan University Kunming China

**Keywords:** Asia, Dry-hot valley, endemism, limestone grassland, phenology, procumbent shrub

## Abstract

*Isodonxiaoluzhiensis*, a new species of the tribe Ocimeae in family Lamiaceae, is described and illustrated. The new species is known only from the type locality, Xiaoluzhi village in Luzhijang dry-hot valley of Yimen County, central Yunnan, southwest China. It is characterized by having a procumbent habit, gracile stems and branches, relatively small leaves and flowers, and the phenology of flowering in winter. The morphological comparisons with its putative closest relatives (*I.adenanthus* and *I.hsiwenii*) are also presented.

## ﻿Introduction

The genus *Isodon* (Benth.) Schrad. ex Spach is a genus of approximately 100 species widely distributed across tropical and subtropical Asia, and with two endemic species in Africa (Wu and Li 1977; Li 1988; Li and Hedge 1994; Mabberley 2008; Zhong et al. 2010; Chen et al. 2019). It was originally placed under the *Plectranthus* L’Hér. as a section (Bentham 1832). Soon after, it was raised to generic rank by Spach (1840). Nevertheless, Spach’s treatment was ignored or overlooked by many later authors (Hasskarl 1842; Bentham 1876; Nakai 1934; Morton 1962), and the name *Rabdosia* (Bl.) Hassk. was widely applied to this genus (e.g. Hara 1972; Li 1975; Wu and Li 1977; Tang and Eisenbrand 1992), until Farr et al. (1979) and Hara (1985) regarded *Isodon* having priority over *Rabdosia*. *Isodon* is now recognized as the only genus in the subtribe Isodoninae (Zhong et al. 2010) and it can be delimited from other genera of the tribe Ocimeae by its bracteolate cymes with a peduncle, actinomorphic or two-lipped (3/2) calyces, strongly two-lipped (4/1) corollas, and stamens with free filaments inserted at the base of the corolla tube (Li 1988; Paton and Ryding 1998; Harley et al. 2004; Chen et al. 2019).

China possesses a rich set of species of *Isodon*, and the center of species diversity of the genus was found in southwest China, especially in the Hengduan Mountains region (Li 1988; Li and Hedge 1994; Yu et al. 2014). The first comprehensive revision of this genus in China was conducted by Wu and Li (1977) for the “Flora Reipublicae Popularis Sinicae”, in which the generic name *Rabdosia.* was applied instead of *Isodon*. Wu and Li (1977) recognized 90 species and 21 varieties in China, and divided these Chinese species into four sections, namely Isodonsect.pyramidium (Benth.) H. W. Li, I.sect.amethystoides (Benth.) H. W. Li, I.sect.Isodon, and I.sect.Melissoides (Benth.) H. W. Li, and the section Isodon was further divided into 10 series. Li and Hedge (1994) reviewed the Chinese species in the *Flora of China*, recognized 77 species in China, 64 being endemics. Recently taxonomic novelties of this genus have been consistently reported from China (Xiang and Liu 2012; Chen et al. 2014, 2016, 2017, 2019, 2021).

In January 2018, during our botanical fieldwork to the Luzhijiang River valley at Yimen County, Yunnan, southwest China, an unknown plant of *Isodon* was encountered and gathered. In 25 September 2021, the same plant was discovered again at the same site. Based on critical comparison with related species, it was confirmed that this plant represents a distinct new species which is described here.

## ﻿Materials and methods

The study followed the normal practice of plant taxonomic survey and herbarium taxonomy. Morphological studies of the new species were based on observation of living plants and specimens housed at YUKU. Digital images of type specimens of genus *Isodon* available at the JSTOR Global Plants (http://plants.jstor.org/), as well as collections housed at CDBI, KUN, PE, PYU and YUKU, were extensively examined and compared with the new species. Pertinent taxonomic literature (Wu and Li 1977; Li 1988; Xiang and Liu 2012; Chen et al. 2014, 2016, 2017, 2019, 2021) was extensively consulted. Measurements were carried out under a stereomicroscope (Olympus SZX2, Tokyo, Japan) using a ruler and a metric vernier caliper.

## ﻿Taxonomy

### ﻿ Isodon xiaoluzhiensis

Taxon classificationPlantaeLamialesLamiaceae

Huan C. Wang & Shi Gang Li sp. nov.

C5886DCD-1555-52E9-B3BA-3F13591C5AAA

urn:lsid:ipni.org:names:77335145-1

Figs 1 , 2 , 3 , 4

#### Type.

China. Yunnan Province: Yimen County, Luzhi Town, Xiaoluzhi village, Maomao Mountain, on limestone grasslands, 24°40'N, 101°57'E, alt. 1300–1400 m, 18 January 2018, *H. C. Wang et al. YM8034* (Holotype: YUKU!; isotype: YUKU!, PE!, KUN!).

#### Diagnosis.

*Isodonxiaoluzhiensis* is most similar to *I.adenanthus* (Diels) Kudô in having similar flower shape, but it clearly differs from the latter in its procumbent (vs. erect or ascending in *I.adenanthus*) habit, stems and branches woody (vs. non-woody) with densely white glandular puberulent (vs. densely retrorse gray pubescent), leaves usually narrowly ovate to rhomboid, rarely lanceolate (vs. rhombic-ovate to ovate-lanceolate), small, 0.8–1.4 cm long (vs. 1.5–6.5 cm long), 0.2–0.5 cm wide (vs. 1.0–2.5 cm wide), teeth of calyx subobtose to subacute (vs. apiculate) at apex, posterior lip of corolla non-spotted (vs. purple spotted). Additionally, *I.xiaoluzhiensis* flowers from November to January, nevertheless *I.adenanthus* usually flowers from March to August.

**Figure 1.**
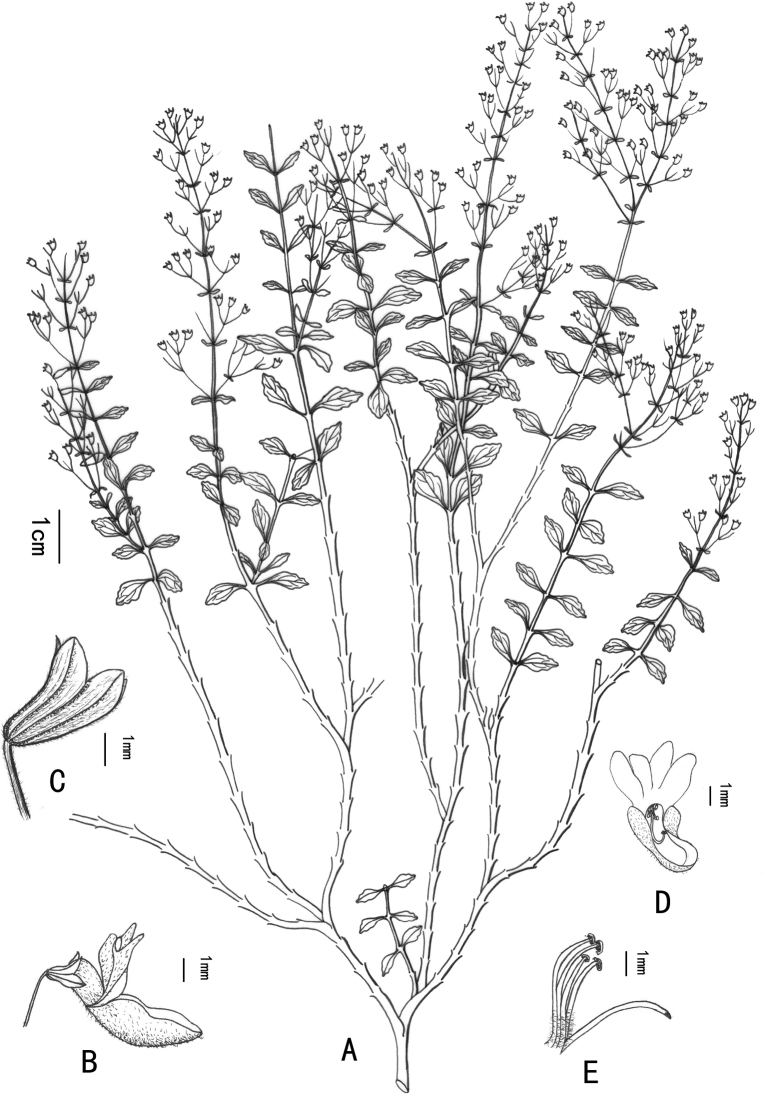
*Isodonxiaoluzhiensis* sp. nov. **A** habit **B** flower (side view) **C** calyx **D** corolla (vertical view) **E** style and stamens.

#### Description.

Small shrubs or subshrubs. Stems woody, procumbent, branched; branches subterete to terete, usually grey, irregularly decorticate, glabrescent; branchlets obtusely quadrangular, purplish, densely white glandular-puberulent. Leaves opposite; petiole 1–3 mm long, rarely subsessile; leaf blades papery or thinly coriaceous, usually narrowly ovate to rhomboid, sometimes lanceolate, 0.8–1.4 cm long, 0.2–0.5 cm wide, apex acute, base cuneate to narrowly cuneate, adaxial surfaces green in young leaves, purplish black when ageing, with pellucid glands, abaxial surfaces gray-green, densely white glandular-puberulent; margin coarsely serrated, with 1–4 teeth on each margin, sometimes entire; veins adaxially depressed, abaxially prominent, with coarse short white hirsute puberulent, lateral veins 2–3 paired. Inflorescences terminal racemose or paniculate, composed of dichasial cymes. Peduncles of cymes gracile, 4–5 mm long, white glandular-puberulent; lax usually with 3–5 flowers; bracts ovate small, subsessile. Flowers small, pedunculate; pedicels gracile, with white glandular-puberulent, 4–5 mm long. Calyx campanulate, conspicuously 10-veined, densely white hirsute outside, 2–3 mm long, 2.0–2.5 mm wide, inconspicuously 2-lipped; posterior lip 3-toothed, subequal, ovate, ca. 1 mm long, usually subobtuse to subacute at apex; anterior lip 2-toothed, equal, ovate, 1.0–1.2 mm long, subobtuse at apex; tube declinate, usually 2 mm long. Corolla purple or light purple, bilabiate, 4–5 mm long; tube tubular, geniculate at base, ca. 2 mm long, densely pubescent outside; posterior lip strongly reflexed, 4-lobed, apex round; anterior lip concave, navicular, obviously longer than the tube, 2.5–3.0 mm long. Stamens 4, didynamous, exserted, inserted at bottom of corolla tube; filaments slender, white villous at base, 5–6 mm long; anthers bluish-purple, elliptic, versatile. Pistil 1, style exserted,7–8 mm long, slightly longer than filaments; ovary superior. Nutlets nearly ovoid, glabrous, sparsely glandular.

**Figure 2.**
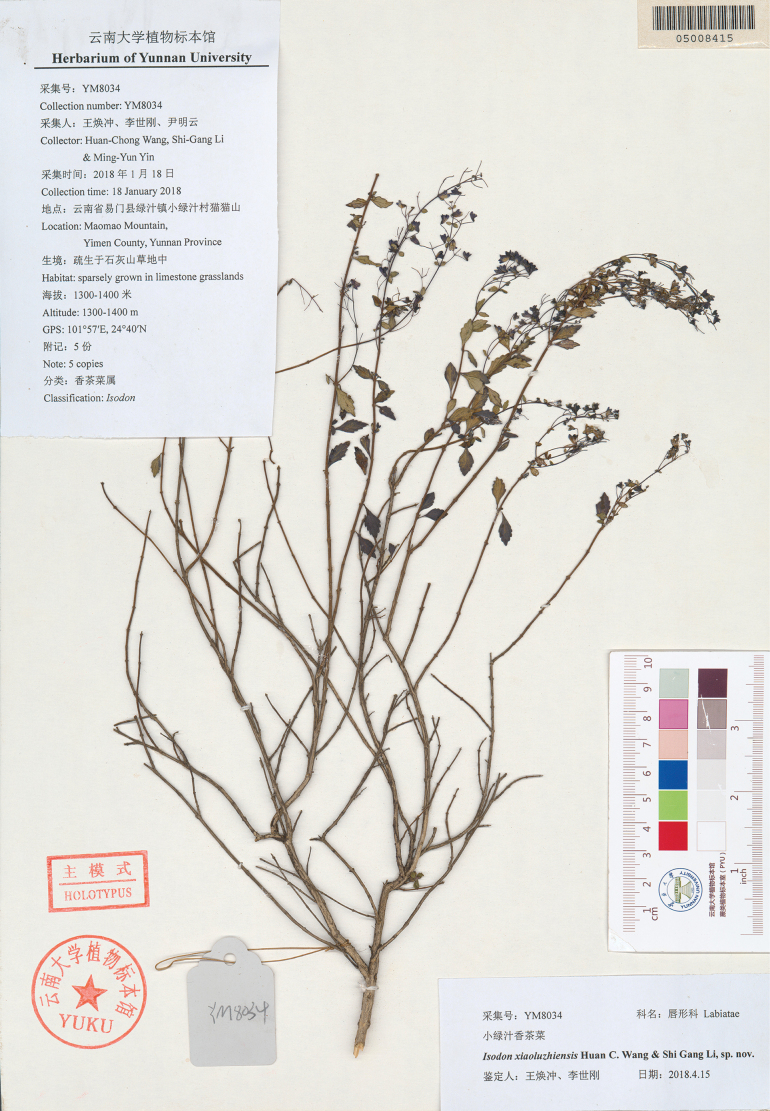
Holotype of *Isodonxiaoluzhiensis* (YUKU-05008415).

#### Phenology.

Flowering from November to January, fruiting from December to February.

#### Etymology.

The specific epithet “*xiaoluzhiensis*” is derived from the type locality of the new species, the Xiaoluzhi village, and the Latin suffix -*ensis*, indicating the place of origin or growth.

**Figure 3.**
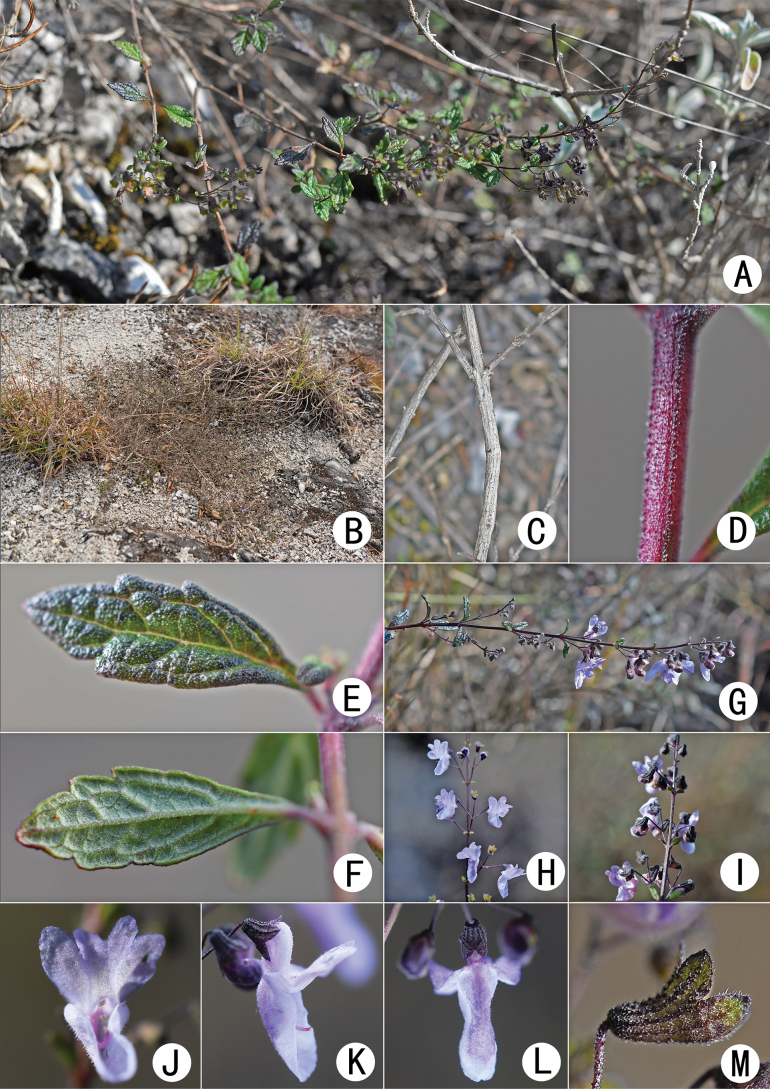
*Isodonxiaoluzhiensis* sp. nov. **A, B** habit **C** perennial stem **D** branchlet **E** adaxial surface of leaf **F** abaxial surface of leaf **G–I** inflorescence **J** corolla (front view) **K** flower (lateral view) **L** corolla (rear view) **M** calyx.

#### Vernacular name.

Chinese mandarin: xiao lu zhi xiang cha cai (小绿汁香茶菜).

#### Distribution and ecology.

According to the present investigations, *I.xiaoluzhiensis* is only found in its type locality, i.e. Xiaoluzhi village of Luzhijiang valley, Yimen County, Yunnan Province, southwest China, where the climate is seasonally hot and arid. *Isodonxiaoluzhiensis* occurs in the limestone grasslands between 1,300 m and 1,400 m elevation. In the type locality, its association mainly includes *Dodonaeaviscosa* (L.) Jacq. (Sapindaceae), *Indigoferavallicola* Huan C.Wang et Jin L. Liu (Leguminosae) (a new species discovered by Liu et al. (2022)), *Duhaldealachnocephala* Huan C. Wang et Feng Yang (Compositae) (an endemic species of Luzhijiang valley discovered by Yang et al. (2022)), *Selaginellapulvinata* (Hook. et Grev.) Maxim (Selaginellaceae), *Pterygiellaluzhijiangensis* Huan C. Wang (Orobanchaceae) and *Onosmadecastichum* Y. L. Liu (Boraginaceae). Among them, the type localities of *I.vallicola*, *D.lachnocephala*, *P.luzhijiangensis*, *O.decastichum* are also in Xiaoluzhi of the Luzhijiang valley (Qiao et al. 2018; Liu et al. 2022; Yang et al. 2022).

**Figure 4.**
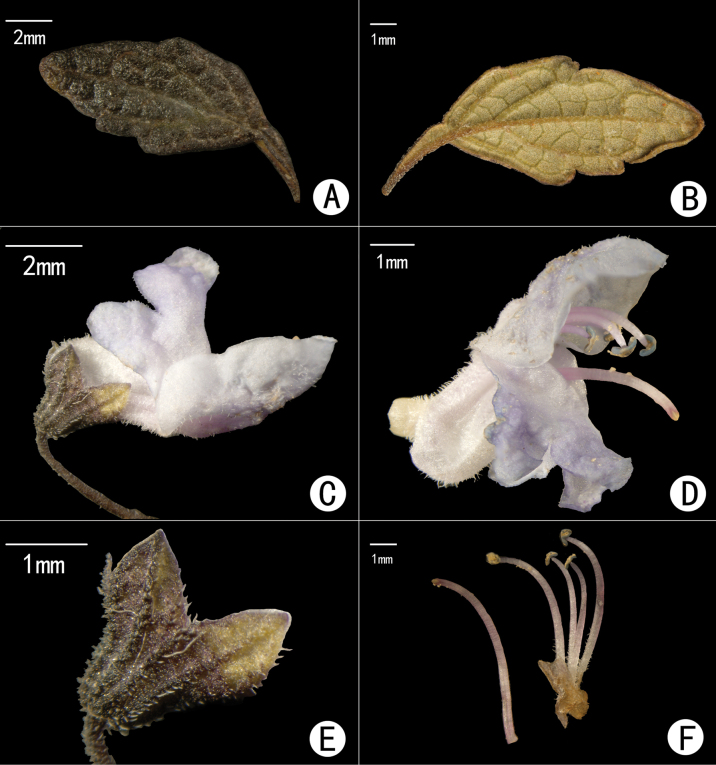
*Isodonxiaoluzhiensis* sp. nov. **A** adaxial surface of leaf **B** abaxial surface of leaf **C** flower (lateral view) **D** flower without calyx **E** calyx **F** pistil and stamens.

#### Preliminary conservation status.

*Isodonxiaoluzhiensis* is a rare species with a restricted distribution and small population size. It is only known from in the upstream region of the Luzhijiang River in the Yimen county, which is no protected area covering. The total population size is estimated at less than 50. According to the IUCN Standards and Petitions Subcommittee (2019), this new species should be considered as "Critically Endangered" (CR).

#### Additional specimens examined

**(paratypes): China. Yunnan**: Yimen County, Luzhi town, Xiaoluzhi village, Maomao Mountain, elev. ca. 1390 m, 25 Sep. 2021, *H. C. Wang et al. YM14638* (YUKU).

#### Discussion.

*Isodonxiaoluzhiensis* exhibits all the characteristics of *Isodon*, but having a procumbent habit, relatively small leaves, and the phenology of flowering in winter can easily differentiate it from other species in the genus. Especially, the procumbent habit is rare in the entire genus *Isodon*, just *I.xiaoluzhiensis* and *I.hsiwenii*, nevertheless the latter is sometimes arcuate. *Isodonxiaoluzhiensis* is very similar to *I.adenanthus* (Diels) Kudô in terms of flower shape and size, but it clearly differs from the latter by its stem woody (vs. non-woody), procumbent (vs. erect or ascending), highly (vs. sparsely) branched, densely white glandular puberulent (vs. densely retrorse gray pubescent), leaves usually narrowly ovate to rhomboid (vs. rhombic-ovate to ovate-lanceolate), papery or thinly coriaceous (vs. herbaceous), small, 0.8–1.4 cm long (vs. 1.5–6.5 cm long), 0.2–0.5 cm wide (vs. 1–2.5 cm wide), teeth of calyx subobtuse to subacute (vs. apiculate) at apex, posterior lip of corolla non-spotted (vs. purple spotted).

*Isodonxiaoluzhiensis* is somewhat close to *I.hsiwenii* Y. P. Chen et C. L. Xiang in sharing relatively small leaves and procumbent stems. However, *I.xiaoluzhiensis* diffeers from *I.hsiwenii* by its main stems up to 60 cm long (vs. up to 100 cm for *I.hsiwenii*), leaves adaxially green or purplish black with pellucid glands (vs. dark green, densely puberulent and colorless glandular), leaves abaxially gray-green and densely white glandular-puberulent (vs. light green, densely puberulent colorless glandular on both sides), calyx purple with few green (vs. green outside), veins densely white hirsute outside (vs. densely purplish puberulent on veins), calyces teeth at apex subobtuse to subacute (vs. acute). Additionally, the habitats of these two species are distinctly different and non-overlapping. *Isodonhsiwenii* is only known from northeast Yunnan, situated in Jinshajiang River basin, and grows on stony slopes at an altitude of approximately 1 750 meters. Conversely, *I.xiaoluzhiensis* is discovered in Central Yunnan, located within the Honghe River basin, and inhabits the limestone grasslands between 1300 m and 1400 m at elevation. A morphological comparison of *I.xiaoluzhiensis* with *I.adenanthus* and *I.hsiwenii* is provided in Table 1.

**Table 1.** A morphological comparison of *Isodonxiaoluzhiensis* with its morphological relatives.

Characters	Species		
	*I.xiaoluzhiensis*	*I.adenanthus*	*I.hsiwenii*
Habit	Shrub or subshrub	herb	shrub
Stems	procumbent	erect or ascending	Procumbent, somewhat arcuate
Stems indumentum	densely white glandular puberulent	densely retrorse gray pubescent	densely purplish puberulent
Stems length (cm)	up to 60	15–40	up to 100
Leaves shape	narrowly ovate to rhomboid	rhombic-ovate to ovate-lanceolate	rhombic-ovate
Leaves size (cm)	0.8–1.4 × 0.2–0.5	1.5–6.5 × 1.0–2.5	1.0–2.0 × 0.5–1.0
Leaves adaxially	green or purplish black with pellucid glands	scattered yellowish glandular	dark green, densely puberulent and colorless glandular
Leaves abaxially	gray-green and densely white glandular-puberulent	white pilose, densely white pubescent on veins	light green, densely puberulent colorless glandular on both sides
Lateral veins	2–3 paired	3–4 paired	2–3 paired
Calyces size (mm)	2–3 × 2–2.5	2–3 × 2–4	2–4 × 2–4
Calyces teeth at apex	subobtuse to subacute	apiculate	acute
Corollas color	light purple	blue, purple, pink, or white	white to light purple
posterior lips of corolla	non-spotted	purple spotted	non-spotted
Phenology	fl. Nov.–Jan., fr. Dec.–Feb.	fl. Jun.–Aug., fr. Jul.–Sep.	fl. Sep.–Nov., fr.Nov.–Dec.

## Supplementary Material

XML Treatment for Isodon xiaoluzhiensis

## ﻿Additional information

### Author ORCIDs

Shi-Gang Li https://orcid.org/0009-0000-4041-6236

Qiang-Chun Huang https://orcid.org/0009-0001-9918-9899

Shao-Yun Liu https://orcid.org/0009-0001-6524-7632

Chun-Lei Xiang https://orcid.org/0000-0001-8775-6967

Huan-Chong Wang https://orcid.org/0000-0001-8562-8849

### Data availability

All of the data that support the findings of this study are available in the main text.
